# Swipe, tap, read? Unveiling the effects of Touchscreen devices on Emergent Literacy Development in preschoolers

**DOI:** 10.1186/s12887-023-04450-y

**Published:** 2023-12-09

**Authors:** Jariyaporn Chowsomchat, Sasivara Boonrusmee, Therdpong Thongseiratch

**Affiliations:** 1 Department of Pediatrics, Faculty of Medicine, Prince of Songkla University, Songkhla, Thailand; 2 Songklanagarind ADHD Multidisciplinary Assessment and care Team for quality Improvement (SAMATI), Child Development Unit, Department of Pediatrics, Faculty of Medicine, Prince of Songkla University, Songkhla, Thailand

**Keywords:** Touchscreen devices, Emergent literacy, Early literacy, Electronic media, Preschool children, Thai, Phonological awareness

## Abstract

Emergent literacy skills are vital for children’s reading and writing development. While touchscreen devices have been linked to enhanced emergent literacy in developed countries, their impact in low- and middle-income countries (LMICs), with limited access to quality apps, is underexplored. Thailand, classified as an upper-middle-income country, presents a unique context with its specific challenges in educational technology, which have not been extensively studied. This study examined the relationship between touchscreen device usage and emergent literacy development in Thai preschool children. Using a cross-sectional design, we analyzed data from 317 Thai children aged 5–6 years, assessing their emergent literacy skills and examining the association with touchscreen device usage through logistic regression analysis. Our findings showed that 79.5% of participants engaged with touchscreen devices, and there was an observed trend suggesting that exclusive tablet users might exhibit enhanced phonological awareness, letter naming, and rapid automatized naming skills. However, these potential improvements did not reach statistical significance when primary caregiver characteristics were taken into account. Our findings highlight the complexity of this relationship and underscore the need for further research to elucidate the potential influences of application quality and screen time engagement on emergent literacy, particularly in LMICs.

## Introduction

In contemporary society, technology exerts significant influence on children, as evidenced by the remarkable surge in touchscreen device usage among them [1, 2]. Approximately one-third of American preschoolers utilize touchscreen devices for a daily duration of two hours [3]. The advantages derived from employing touchscreen devices are contingent upon the child’s age, individual attributes, developmental phase, parental scaffolding presence, and media content [4]. Touchscreen devices serve as instruments for play, communication, and expression in preschool environments. Several studies investigating the impact of touchscreen devices on early childhood learning have demonstrated improvements in reading and writing skills, vocabulary acquisition, and print knowledge [5, 6]. Nevertheless, the educational merits of touchscreen devices have been observed to be relatively restricted [7, 8]. Additionally, certain drawbacks of touchscreen devices have been identified, such as elevated media usage associated with physical (obesity and cardiovascular risk) and psychological (psychosocial development and self-regulation) health complications [9–13]. Given the escalating prevalence of touchscreen devices among preschoolers, it is imperative to scrutinize the connection between touchscreen device utilization and emergent literacy.

## Touchscreen devices

Touchscreen devices, though often discussed as a collective term, have noteworthy differences in design, functionality, and usage patterns, especially when it comes to children’s educational experiences [5, 6]. Tablets, for instance, generally have a larger screen real estate compared to mobile phones, allowing for more interactive and detailed educational applications. The size and format of tablets often promote a more collaborative use, where parents or caregivers can jointly engage with children, fostering shared learning experiences [5, 6]. On the other hand, mobile phones, primarily designed for personal use, tend to facilitate more isolated interactions and might be predominantly used for short-span entertainment or communication purposes [4]. Recognizing these inherent differences is essential, as they might lead to varied outcomes in emergent literacy development.

## Emergent literacy

Emergent literacy encompasses a child’s pre-existing knowledge of reading and writing skills before formal instruction in reading and writing commences. These skills are essential for future reading and writing expertise [14]. Individual disparities in reading and writing are predicted by emergent literacy skills such as phonological awareness, letter knowledge, and rapid automatized naming, irrespective of linguistic variation [15, 16]. Phonological awareness refers to the ability to identify and manipulate phonemes, or individual sounds, within words. This foundational skill is pivotal for reading and spelling. Letter knowledge encompasses the recognition and identification of individual letters and their associated sounds, serving as a bridge between phonological awareness and actual reading. Lastly, rapid automatized naming describes the ability to quickly verbalize a series of familiar items, like numbers or colors; this ability has been linked to fluency in reading as it reflects the efficiency of accessing phonological information [14]. Each of these constructs plays a significant role in the broader landscape of emergent literacy development. Several prior studies have underlined the predictive nature of these three parameters concerning future reading proficiencies. These parameters, hence, not only provide a snapshot of a child’s current literacy skills but also offer insights into their future reading trajectory [14–16].

A recent study discovered a correlation between phonological awareness and orthographic knowledge in readers of alphasyllabic languages, which are spoken in South and Southeast Asia, Ethiopia, Northern Africa, and certain regions of North America [17]. In Thai, an alphasyllabic language, phonological awareness, letter knowledge, and rapid automatized naming significantly predict early-stage reading and spelling achievement [18, 19]. Emergent literacy skills develop within the context of various direct and indirect environmental factors, such as parental interaction, the quantity of books in the home, and media usage [20, 21]. The caliber of the literacy-related environment parents provide for their children significantly impacts the evolution of children’s emergent literacy skills [22, 23].

### The association of touchscreen device use and emergent literacy

Several prior studies have identified a relationship between touchscreen device usage and emergent literacy [7]. Neumann’s 2014 study explored the relationship between touchscreen tablet usage and emergent literacy skills in Australian preschoolers aged 3–5 years. The study highlighted that children with increased access to touchscreen tablets exhibited better letter sound and name writing abilities, suggesting that tablet usage could positively contribute to the development of emergent literacy skills. However, the research did not find a direct correlation between the amount of time spent on tablets and emergent literacy skills. This indicates that factors other than mere tablet exposure, such as the quality of content, parental involvement, and children’s individual learning styles, may play an essential role in the development of these skills. Neumann’s study emphasizes the importance of considering various factors when assessing the impact of touchscreen tablets on young children’s literacy development [24]. Hutton et al.‘s 2020 study investigated the relationship between screen-based media usage and emergent literacy skills in American preschoolers aged 3–5 years. Contrary to Neumann’s 2014 study, Hutton et al. found that increased use of screen-based media was associated with lower emergent literacy skills in young children. This study suggests that excessive exposure to screen-based media might have a negative impact on the development of these crucial early literacy abilities. However, it is essential to consider that the quality of content, parental involvement, and the context in which the screen-based media is used could contribute to the observed outcomes [25].

The diverse outcomes in previous research on emergent literacy skills suggest a significant role for external variables, from environmental factors like home literacy environments to genetic influences. Xie et al.’s 2018 meta-analysis, which examined the effects of touchscreen devices on young children’s learning outcomes, is particularly enlightening in this regard. Their study, involving 36 empirical articles and 4,206 participants, found a medium overall effect size (d = 0.46) in favor of touchscreen devices for enhancing learning outcomes in children, with 82.3% of effect sizes being positive. Notably, the benefit of using touchscreen devices was more pronounced in learning STEM subjects compared to non-STEM areas, highlighting the suitability of these devices for certain types of knowledge. Age was a significant moderator; older children showed more substantial gains, indicating a developmental aspect in digital learning readiness. Furthermore, when compared to various baseline conditions like traditional classroom teaching or other forms of digital learning, touchscreen devices showed a more substantial impact on learning. These findings emphasize that while touchscreen devices can enhance learning, their effectiveness is highly dependent on factors like the age of the child, the subject matter, and the learning environments [26].

While research from high-income countries highlights positive associations between touchscreen use and emergent literacy [7, 24], the situation in low- and middle-income countries (LMICs) offers a unique perspective [26]. In many LMICs, despite the growing prevalence of these devices, there is limited access to high-quality educational applications [27–30]. Kim’s 2021 systematic review and meta-analysis found that among 36 reviewed educational apps, only 3 were from LMICs [27], highlighting a stark deficiency in effective educational resources, particularly in countries like India [28], Indonesia [29], and Malawi [30]. This evidence directly supports the concerns raised about the availability and quality of educational applications in LMICs. This lack of access can be attributed to factors such as cost barriers, limited localized content [28, 29], and lack of awareness or knowledge about quality apps among caregivers and educators [29, 30]. This disparity raises questions about the potential impact of touchscreen devices on emergent literacy skills in preschool children from LMICs, an area that this study seeks to explore.

According to Thailand’s Ministry of Public Health, children who have primary caregivers that utilize high-quality electronic device applications and actively engage with them during device usage tend to exhibit better developmental outcomes. On the other hand, children who do not use electronic devices at all or rely on low-quality applications often show inferior developmental outcomes [31]. These findings emphasize the importance of taking into account various factors, such as content quality, parental involvement, and context, when assessing the impact of touchscreen devices on children’s emergent literacy skills. This understanding can help parents, educators, and policymakers make informed decisions about incorporating technology into early childhood education to best support children’s learning and development.

Given the widespread use of touchscreen devices among families with preschoolers [3, 27], it is crucial to investigate the associations between emergent literacy skills and touchscreen device usage. The primary aim of this study was to explore the relationship between touchscreen device use and emergent literacy in preschool children. Previous research in this area has some limitations, as it did not adequately control for factors that may influence emergent literacy. Consequently, our study sought to identify additional factors, such as information related to the primary caregiver and the child, which could impact a child’s emergent literacy skills. Furthermore, this study focused on Thai preschool children, making it the first to investigate this topic in a developing country and the first to explore an alphasyllabary—a language that represents sounds at both syllable and phoneme levels [17].

Thailand, classified as an upper-middle-income country, exemplifies a unique context with its distinct challenges in educational technology, which have not been comprehensively explored. The nation showcases a blend of urban and rural landscapes, leading to varied access and quality in educational resources. In urban areas, there is potential access to high-quality educational applications, while rural regions may face significant constraints. This disparity is not solely due to cost implications but also stems from differences in infrastructure, such as more reliable internet connectivity and higher levels of digital literacy found in urban settings [28]. Additionally, urban residents often have greater awareness and exposure to digital educational resources, contributing to this unequal access [29]. This disparity highlights the need for an inclusive and equitable approach to education, aligning with the Sustainable Development Goals (SDGs) that advocate for quality education for all. Our study is situated within this complex environment, aiming to unravel the impact of touchscreen device usage on emergent literacy and provide valuable perspectives to the global research community.

## Methods

### Research design

In this study, we utilized a cross-sectional analysis derived from the Thai Emergent Literacy for Predicting Dyslexia (TED) longitudinal study to investigate the relationship between touchscreen device use and emergent literacy among preschool children. The data used for this study represents a cross-sectional analysis focused on the T1 time point of our ongoing longitudinal research. Data collection for this specific time point took place before the emergence of the COVID-19 pandemic. Specifically, our data collection dates for the T1 phase spanned from November 2018 to January 2019. As such, the results and observations made in this study are uninfluenced by the potential impacts, behavioral changes, or restrictions introduced during the COVID-19 period. The research focused on young children attending both public and private kindergarten schools in a large city in Southern Thailand. This urban setting in an LMIC provides a distinctive context where access to digital educational resources is likely more prevalent compared to rural areas, yet still faces the broader challenges typical of LMICs, such as limited resources and varied levels of digital literacy among the population. The study was conducted in compliance with the ethical guidelines set forth by the Declaration of Helsinki. Additionally, the research protocol received approval from the Institutional Review Board (IRB) of the Faculty of Medicine, Prince of Songkla University, Thailand (Project identification code: HREC 16-1256342). Ethical approval for the study was granted on July 13, 2018.

### Participants

The study participants were selected from 10 primary schools with kindergartens in Hatyai, Songkhla, a bustling urban city in Thailand, using a non-probability sampling method. To qualify for the study, children needed to meet the following criteria: (a) aged between 5 and 6 years; (b) not having any diagnosed developmental delays or intellectual disabilities, as confirmed by school records and parental reports; (c) informed consent provided by parents; and (d) belonging to families using the Thai language. Children from bilingual households were specifically excluded to control for potential variations in language acquisition and literacy development. Bilingualism can introduce complexities in the assessment of emergent literacy skills, as children might display varying proficiency levels in each language or use different linguistic strategies. By focusing on monolingual Thai households, the study aimed to ensure a more homogeneous linguistic background, thereby allowing for clearer interpretation of the effects of touchscreen device usage on emergent literacy. Prior to the children’s involvement in the study, their parents signed informed consent forms, ensuring that they were aware of the study’s purpose and their children’s participation.

### Measurements

In the questionnaire provided to the parents, touchscreen device use was measured through a series of yes/no questions related to specific devices their child interacted with. This encompassed a range of devices including mobile phones, tablets, televisions, computers, and video games. For instance, parents were posed questions like, ‘Does your child use a mobile phone?‘ and ‘Does your child play video games?‘. This approach allowed us to obtain a straightforward understanding of the diversity of digital interfaces the children were exposed to.

Emergent literacy skills were evaluated with the Rama Pre-Read (RPR) software program. The test’s main objective is to evaluate in the age range of 60–84 month children in the various domains. In the present study, we assessed on emergent literacy skills using the following 3 tasks: phonological awareness, rapid automatized naming and letter naming.

### Phonological awareness tasks

The initial phoneme matching task in the RPR program was used to assess phonological awareness. The children were given three practice trials before testing; after that, it was proceeded with 10 test items in this task. For each item, 4 picture drawings were shown to the child. One of the pictures was the stimulus, and the other 3 were the answer choices. All pictures depicted monosyllabic and meaningful nouns familiar to Thai preschool children [19]. The child psychologist, who was the tester, verbally named all 4 pictures in each item and asked the child to identify which picture from the choices had the same initial phoneme as the stimulus picture. For example, the stimulus picture was “nok” (“nok”means bird in English), and the 3 choices were “nhu” (“nhu” means rat in English), “lor” (‘lor’ means wheel in English), and “khon” (“khon” means human in English). If the child pointed to the picture indicating the word “nhu” when the stimulus picture was “nok”, s/he would receive 1 point. The score was the aggregate of the scores obtained from each correct answer. The Cronbach’s alpha reliability coefficient in our sample was 0.96.

#### Rapid automatized naming task

In this test, the child was shown a laptop screen containing five rows of familiar letters, with each row containing the same five letters arranged in a different order. Before the timed test, the children were asked to name all five letters in a practice trial to ensure they could name the letters correctly. They were then asked to identify and read aloud the 25 letters in the specified order as quickly as possible [19]. The score for this task represented the total time used to complete the task. This task’s test-retest reliability score was 0.91.

### Letter-naming task

The letter-naming task in the RPR program was used to assess the participants’ letter knowledge based on how many of the 44 letters of the Thai alphabet they could name. In the Thai version of this program used in our study, the children were presented with one letter at a time on a single page and then asked to name it. If the child named the letter correctly, s/he gained one point. The letters were arranged by letter familiarity, starting with the first and most familiar letters [19]. The order of these letters is fixed in the RPR software, and the maximum score for this is 44. The Cronbach’s alpha reliability coefficient in our sample was 0.94.

### Procedure

Prior to testing, informed consent was secured from all participating parents. Subsequently, parents filled out a comprehensive written questionnaire capturing details about their family’s demographic background as well as specifics regarding the child’s touchscreen device usage – type and duration. Each child was then individually evaluated by a child psychologist with substantial expertise in psychological assessments. These assessments were conducted in a distraction-free room at the participants’ respective schools during regular school hours. During the emergent literacy tasks, children’s responses were both recorded and monitored in real-time by the child psychologist to ensure accuracy in capturing the child’s responses. This approach allowed for immediate clarification or repetition of instructions if necessary. The entire assessment spanned roughly 15 min per child. To ensure the validity and reliability of our assessment, while also catering to the attention span of 5–6 year-olds, the order of the tests was randomized for each child. Given the 15-minute session duration, our testing software employed a game-like interface specifically designed for children, ensuring that the tasks remained engaging and age-appropriate. Despite the short testing duration, we still incorporated brief relaxation intervals between tasks to help maintain children’s interest and motivation. This child-centric approach aimed to capture accurate and consistent responses without overwhelming our young participants.

### Data analysis

All data were analyzed using the R software, version 4.1.1. Initial steps involved data cleaning to ensure its quality adhered to established research practice guidelines. Descriptive statistics (frequencies, percentages, means, and standard deviations) were utilized to provide insights into children’s gender, the primary type of caregiver, and the caregiver’s age, education, and occupation. To ascertain differences between study groups, the Chi-square and Fisher’s exact tests were applied.

For the purpose of this study, participants were initially classified into two primary groups: ‘use’ and ‘non-use’ based on their engagement with touchscreen devices. The ‘use’ group encompasses children who interacted with either tablets or mobile phones or both, while the ‘non-use’ group consists of children who didn’t use any touchscreen devices. Delving deeper within the ‘use’ group, participants were further categorized into ‘tablet-only’, ‘mobile-only’, and ‘both’ subgroups. It’s crucial to highlight that this classification specifically pertains to the usage of tablets and mobile phones. Other electronic devices, such as TVs or computers, were not considered in this differentiation. The rationale behind this segmentation is based on the distinct usability and applications that tablets and mobile phones present for children.

Logistic regressions were applied to probe the possible effect of touchscreen device use on each emergent literacy skill. Three models of logistic regression were employed. In Model 1, we did not adjust for any covariable. The univariate analysis indicated that caregiver education was the only factor statistically significantly linked to emergent literacy, leading to its inclusion as an adjustment in Model 2. Model 3 incorporated all variables enumerated in Table 1. This approach was taken due to concerns about potential selection bias, which might skew the significance of the outcomes derived from the univariate regression analysis. P-values below 0.05 were considered statistically significant.

## Results

### Baseline characteristics of children and their families

Of the 317 children, 252 (79.5%) were placed in the use group and 65 (20.5%) in the non-use group. Table 1 shows the differences in the characteristics of the children and their families between the two groups. The children in both groups were not different in terms of child’s gender (χ^2^ = 564.72, *p* < 0.001) and age of caregivers (χ^2^ = 564.722, *p* < 0.001). However, the variables of the type of main caregiver (χ^2^ = 564.722, *p* < 0.001), caregiver educational level (χ^2^ = 564.722, *p* < 0.001), and primary caregiver occupation (χ^2^ = 564.722, *p* < 0.001) were statistically different between the touchscreen device use and non-use groups. Parents were the main caregivers in the use group more frequently than those in the non-use group.

**Table 1:** Baseline characteristics of children and their families

	Use (%)	Non-use (%)	Total (%)	*P* value	Test
**Variable**	252	65	317		
**Child gender**				0.071	Chi-square
**Male**	131 (52)	25 (38.5)	156 (49.2)		
**Female**	121 (48)	40 (61.5)	161 (50.8)		
**Caregiver age (yrs.)**				0.145	Chi-square
**15–25**	17 (6.8)	5 (7.8)	22 (7)		
**26–40**	145 (58.2)	29 (45.3)	174 (55.6)		
**41–50**	68 (27.3)	20 (31.2)	88 (28.1)		
**> 50**	19 (7.6)	10 (15.6)	29 (9.3)		
**Caregiver type**				< 0.001	Chi-square
**Parent**	229 (90.9)	43 (66.2)	272 (85.8)		
**Other**	23 (9.1)	22 (33.8)	45 (14.2)		
**Caregiver education**				0.002	Chi-square
**Less than Bachelor’s degree**	141 (57.1)	50 (79.4)	191 (61.6)		
**Bachelor’s degree** **or higher**	106 (42.9)	13 (20.6)	119 (38.4)		
**Caregiver occupation**				0.034	Fisher’s exact
**Employee**	121 (49)	35 (56.5)	156 (50.5)		
**Business owner**	88 (35.6)	20 (32.3)	108 (35)		
**Government officer**	21 (8.5)	0 (0)	21 (6.8)		
**None**	17 (6.9)	7 (11.3)	24 (7.8)		

### Emergent literacy score by groups

Table 2, presenting mean scores and adjusted Beta coefficients for phonological awareness, rapid automated naming time, and letter naming. In unadjusted analyses, tablet-only users demonstrated higher phonological awareness, quicker rapid automated naming times, and better letter naming scores compared to non-users. However, these associations diminished and became non-significant after adjusting for the educational level of the primary caregiver and other demographic factors.

Mobile-only and users of both device types did not show significant differences in emergent literacy skills compared to non-device users across all models. These findings indicate that while there is a preliminary association between tablet use and improved literacy skills, this relationship is not maintained when accounting for familial and demographic variables.

**Table 2:** Phonological awareness score, rapid automated naming time, and letter naming score by groups

Characteristic	Mean ± SD	Model 1: Unadjusted Beta (95% CI)	*P*-value	Model 2: Adjusted Beta (95% CI)	*P*-value	Model 3: Adjusted Beta (95% CI)	*P*-value
*Phonological awareness score (points)*							
None	4.8 ± 1.8	0 (*Reference*)		0 (*Reference*)		0 (*Reference*)	
Tablet-only	6.0 ± 2.5	**1.26 (0.37, 2.14)**	**0.006**	0.60 (-0.22, 1.42)	0.152	0.63 (-0.23, 1.49)	0.154
Mobile-only	4.9 ± 2.3	0.14 (-0.57, 0.84)	0.703	-0.02 (-0.66, 0.62)	0.951	0.04 (-0.63, 0.70)	0.914
Both	5.5 ± 2.4	**0.73 (0.02, 1.43)**	**0.044**	0.12 (-0.54, 0.77)	0.723	0.19 (-0.49, 0.86)	0.591
Remarks		R^2^ = 0.025 F-value = 3.756 df = 3 and 313		R^2^ = 0.196 F-value = 20.2 df = 4 and 312		R^2^ = 0.191 F-value = 9.264 df = 9 and 307	
*Rapid automated naming time (minutes)*							
None	1.03 ± 0.52	0 (*Reference*)		0 (*Reference*)		0 (*Reference*)	
Tablet-only	0.73 ± 0.41	**-0.30 (-0.52, -0.09)**	0.006	-0.17 (-0.38, 0.03)	0.101	-0.14 (-0.35, 0.07)	0.195
Mobile-only	1.01 ± 0.61	-0.02 (-0.19, 0.15)	0.806	0.01 (-0.15, 0.17)	0.885	0.05 (-0.12, 0.21)	0.576
Both	0.91 ± 0.55	-0.12 (-0.29, 0.06)	0.185	0.00 (-0.16, 0.17)	0.958	0.02 (-0.15, 0.19)	0.803
Remarks		R^2^ = 0.021 F-value = 3.203 df = 3 and 309		R^2^ = 0.138 F-value = 13.44 df = 4 and 308		R^2^ = 0.163 F-value = 7.77 df = 9 and 303	
*Letter naming score (points)*							
None	33.3 ± 10.0	0 (*Reference*)		0 (*Reference*)		0 (*Reference*)	
Tablet-only	38.2 ± 6.8	**4.85 (1.51, 8.19)**	**0.005**	2.82 (-0.37, 6.01)	0.084	2.87 (-0.41, 6.15)	0.088
Mobile-only	35.2 ± 8.7	1.84 (-0.80, 4.48)	0.173	1.35 (-1.13, 3.83)	0.287	1.38 (-1.15, 3.90)	0.285
Both	36.7 ± 8.0	**3.34 (0.70, 5.99)**	**0.013**	1.46 (-1.09, 4.00)	0.263	1.78 (-0.80, 4.35)	0.177
Remarks		R^2^ = 0.022 F-value = 3.41 df = 3 and 313		R^2^ = 0.138 F-value = 13.64 df = 4 and 312		R^2^ = 0.170 F-value = 8.188 df = 9 and 307	

Number in bold denotes statistical significance at a 95% level of confidence.

Model 1: Did not adjust for any covariable.

Model 2 adjusted for educational level of primary caregiver.

Model 3 adjusted for educational level of primary caregiver, role of primary caregiver (parents vs. otherwise), occupation of primary caregiver, and sex of child.

### Effect of touchscreen device use on phonological awareness

Table 3 examines the impact of touchscreen device use on children’s phonological awareness. The unadjusted model reveals a notable association for tablet-only use, suggesting improved phonological skills compared to non-use (OR 3.85, 95% CI 1.38–10.71). However, this association becomes less clear in the adjusted models, which account for factors like the caregiver’s educational level. Here, the trend towards better phonological awareness with touchscreen use (mobile-only, tablet-only, or both) is not statistically significant (Mobile-only: OR = 1.41, 95% CI 0.49–4.1; Tablet-only: OR = 2.05, 95% CI 0.62–6.79; Both: OR = 1.16, 95% CI 0.41–3.26). Model fit indices, including log-likelihood and AIC scores, further inform the robustness of these models. For instance, Model 1 (Unadjusted) has a log-likelihood of -151.3092 and AIC of 310.6184, while Model 3 (Fully adjusted) shows − 123.728 and 267.456, respectively. Lower AIC values in adjusted models indicate a better fit but also highlight the influence of various background factors on the relationship between touchscreen device use and phonological awareness.

**Table 3:** Association between touchscreen device usage and phonological awareness (> 7 or high relative awareness vs. ≤7 or average-to-low awareness)

Device Usage	Average-to-low awareness (score ≤ 7)	High awareness (score > 7)	Model 1: Unadjusted OR (95% CI)	*P*-value (LR-test)	Model 2: Adjusted OR (95% CI)	*P*-value (LR-test)	Model 3: Adjusted OR (95% CI)	*P*-value (LR-test)
**None (n = 65)**	58 (89.2%)	7 (10.8%)	1.0 (*Ref.*)		1.0 (*Ref.*)		1.0 (*Ref.*)	
**Mobile only (n = 106)**	88 (83.0%)	18 (17.0%)	1.69 (0.67, 4.31)	0.256	1.46 (0.53, 4.04)	0.457	1.41 (0.49, 4.1)	0.519
**Tablet only (n = 41)**	28 (68.3%)	13 (31.7%)	**3.85 (1.38, 10.71)**	0.008	2.11 (0.68, 6.53)	0.190	2.05 (0.62, 6.79)	0.236
**Both (n = 105)**	82 (78.1%)	23 (21.9%)	2.32 (0.94, 5.78)	0.057	1.21 (0.44, 3.28)	0.709	1.16 (0.41, 3.26)	0.785
**Remarks**			**Log-likelihood = -151.3092** **No. of observations = 317** **AIC value = 310.6184**		**Log-likelihood = -125.7414** **No. of observations = 317** **AIC value = 261.4829**		**Log-likelihood = -123.728** **No. of observations = 317** **AIC value = 267.456**	

Number in bold denotes statistical significance at a 95% level of confidence.

Model 1: Did not adjust for any covariable.

Model 2: Adjusted for educational level of primary caregiver.

Model 3: Adjusted for educational level of primary caregiver, family relationship (parent vs. other), occupation of primary caregiver, and sex of child.

### Effect of touchscreen device on rapid automatized naming

Table 4 assesses the association between touchscreen device usage and rapid automatized naming (RAN) time in children. In the unadjusted model, the tablet-only group showed a significant positive effect on RAN time, suggesting faster performance compared to the non-use group (OR 2.96, 95% CI 1.32–6.66). This implies that tablet usage may positively influence the speed of naming. However, the significance of this association decreases in the adjusted models. While there is an observable trend where children using touchscreen devices appear to have faster RAN times, these associations do not reach statistical significance in the fully adjusted models (Mobile-only: OR = 1.04, 95% CI 0.51–2.11; Tablet-only: OR = 2.07, 95% CI 0.82–5.23; Both: OR = 1.2, 95% CI 0.58–2.48). The model fit indices, including log-likelihood and AIC values, provide insight into each model’s robustness. Model 1 (Unadjusted) has a log-likelihood of -213.6802 and an AIC of 435.3604. Model 3 (Fully adjusted) shows a log-likelihood of -188.5654 and an AIC of 397.1308, indicating a better fit with the inclusion of additional variables.

**Table 4:** Association between touchscreen device usage and rapid automatized naming time (faster than average or < 60 s vs. as fast as average or slower than average or ≥ 60 s)

Device usage	As fast as average or slower than average (time ≥ 60 s)	Faster than average (time < 60 s)	Model 1: Unadjusted OR (95% CI)	*P*-value (LR-test)	Model 2: Adjusted OR (95% CI)	*P*-value (LR-test)	Model 3: Adjusted OR (95% CI)	*P*-value (LR-test)
**None (n = 65)**	41 (63.1%)	24 (36.9%)	1.0 (*Ref.*)		1.0 (*Ref.*)		1.0 (*Ref.*)	
**Mobile only (n = 106)**	64 (60.4%)	42 (39.6%)	1.12 (0.59, 2.12)	0.725	0.99 (0.5, 1.95)	0.972	1.04 (0.51, 2.11)	0.917
**Tablet only (n = 41)**	15 (36.6%)	26 (63.4%)	**2.96 (1.32, 6.66)**	0.008	1.97 (0.82, 4.72)	0.125	2.07 (0.82, 5.23)	0.120
**Both (n = 105)**	52 (49.5%)	53 (50.5%)	1.74 (0.92, 3.28)	0.083	1.1 (0.55, 2.2)	0.780	1.2 (0.58, 2.48)	0.616
**Remarks**			**Log-likelihood = -213.6802** **No. of observations = 317** **AIC value = 435.3604**		**Log-likelihood = -192.7989** **No. of observations = 317** **AIC value = 395.5978**		**Log-likelihood = -188.5654** **No. of observations = 317** **AIC value = 397.1308**	

Number in bold denotes statistical significance at a 95% level of confidence. Model 1: Did not adjust for any covariable; Model 2: Adjusted for educational level of primary caregiver. Model 3: Adjusted for educational level of primary caregiver, family relationship (parent vs. other), occupation of primary caregiver, and sex of child.

### Effect of touchscreen device use on letter naming

Table 5 investigates the association between touchscreen device usage and letter naming scores in children. In the unadjusted model, children in the tablet-only group demonstrated a significantly higher likelihood of achieving above-average letter naming scores compared to the non-use group (OR 2.36, 95% CI 1.05–5.30), suggesting a positive impact of tablet use on letter naming proficiency. However, when adjusting for additional variables, the strength of this association diminishes. In the fully adjusted models, the trend indicating that children using any touchscreen device might achieve higher letter naming scores is observed, but these associations do not reach statistical significance (Mobile-only: OR = 1.03, 95% CI 0.5–2.13; Tablet-only: OR = 1.78, 95% CI 0.71–4.45; Both: OR = 1.23, 95% CI 0.59–2.56). The model fit indices, including the log-likelihood and Akaike Information Criterion (AIC) scores, provide additional context to the models’ robustness. Model 1 (Unadjusted) shows a log-likelihood of -206.0878 and an AIC of 420.1756, while Model 3 (Fully adjusted) has a log-likelihood of -187.9101 and an AIC of 395.8202. These indices suggest a better fit for the adjusted models, indicating that variables such as the educational level of the primary caregiver and family demographics play a role in the relationship between touchscreen device usage and letter naming scores.

**Table 5:** Association between touchscreen device usage and letter naming score (above average or > 40 letters vs. average or below average or ≤ 40 letters)

Device usage	Average or below average (≤ 40 letters)	Above average (> 40 letters)	Model 1: Unadjusted OR (95% CI)	*P*-value	Model 2: Adjusted OR (95% CI)	*P*-value	Model 3: Adjusted OR (95% CI)	*P*-value
**None (n = 65)**	45 (69.2%)	20 (30.8%)	1.0 (*Ref.*)		1.0 (*Ref.*)		1.0 (*Ref.*)	
**Mobile only (n = 106)**	72 (67.9%)	34 (32.1%)	1.06 (0.55, 2.07)	0.858	0.95 (0.48, 1.91)	0.896	1.03 (0.5, 2.13)	0.942
**Tablet only (n = 41)**	20 (48.8%)	21 (51.2%)	**2.36 (1.05, 5.30)**	0.036	1.64 (0.7, 3.85)	0.255	1.78 (0.71, 4.45)	0.216
**Both (n = 105)**	62 (59.0%)	43 (41.0%)	1.56 (0.81, 3)	0.179	1.07 (0.53, 2.15)	0.848	1.23 (0.59, 2.56)	0.579
**Remarks**			Log-likelihood = -206.0878 No. of observations = 317 AIC value = 420.1756		Log-likelihood = -193.2512 No. of observations = 317 AIC value = 396.5024		Log-likelihood = -187.9101 No. of observations = 317 AIC value = 395.8202	

Number in bold denotes statistical significance at a 95% level of confidence.

Model 1: Did not adjust for any covariable.

Model 2: Adjusted for educational level of primary caregiver.

Model 3: Adjusted for educational level of primary caregiver, family relationship (parent vs. other), occupation of primary caregiver, and sex of child.

## Discussion

The purpose of this study was to extend the current knowledge regarding the relationship between touchscreen device use and emergent literacy in Thai preschool children. The children in the touchscreen device-use group showed significantly higher scores than those in the non-use group for all emergent literacy skills. When comparing the different types of touchscreen devices used (mobile only, tablet only, and both mobile and tablet) and their effects on emergent literacy, it was found that the tablet-only group demonstrated the highest phonological awareness and letter naming scores, as well as the fastest rapid automatized naming time. Upon examining the association between touchscreen device use and phonological awareness, unadjusted models indicated that participants in the tablet-only group had significantly greater odds of higher phonological awareness scores compared to the non-use group. However, in fully adjusted models accounting for primary caregiver characteristics, participants who used any type of touchscreen device demonstrated greater odds of better phonological awareness compared to the non-use group, though this difference was not statistically significant. A similar pattern emerged for letter naming scores and RAN.

These results suggest that, while tablet-only use appears to have the most significant impact on emergent literacy skills, the relationship between touchscreen device use and emergent literacy may not be statistically significant after adjusting for primary caregiver characteristics. This indicates that other factors, such as caregiver involvement and the quality of touchscreen applications, may play a more critical role in influencing a child’s emergent literacy skills.

Our findings, which showed that the tablet-only group had the best emergent literacy outcomes without adjusting for covariates, align with the notion that tablets may be more conducive to learning for young children due to their user-friendly interfaces. However, the study by Pedra et al. (2015) offers a more nuanced perspective on the relationship between touchscreen device interactivity and learning outcomes. Pedra et al. (2015) investigated the impact of high and low levels of interactivity on children’s learning experiences with touchscreen devices. Their results revealed that while devices featuring high interactivity levels (e.g., rotating, dragging, or zooming an object) were more engaging for children, they did not necessarily lead to superior learning outcomes compared to devices with lower levels of interactivity. This finding suggests that the relationship between touchscreen device use and emergent literacy skills is complex and not solely determined by the device type or the level of interactivity it offers. Factors such as the quality and appropriateness of the educational content, the child’s cognitive abilities, and the level of parental involvement during the learning process may play more significant roles in shaping a child’s literacy development [32].

Our findings suggest that primary caregiver characteristics, particularly the educational level, should be taken into account when examining the relationship between touchscreen device use and emergent literacy in preschool children. The finding that the educational level of primary caregivers is an important factor influencing children’s emergent literacy skills is consistent with previous studies. For example, a study conducted in Iran found that mother’s education level was significantly associated with children’s emergent literacy skills [33]. Another study conducted in Thailand found that maternal education and household income were associated with children’s initial phoneme-matching and letter-naming scores [19]. The relationship between primary caregiver characteristics and children’s emergent literacy skills is a well-established area of research. Previous studies have also found that parental education level and household income are associated with children’s language and literacy development [20, 21]. These findings are consistent with the hypothesis that caregivers with higher education levels and more financial resources are better equipped to provide a rich home literacy environment and engage in activities that promote language and literacy skills. It is important to consider the potential indirect and direct effects of primary caregiver characteristics on children’s emergent literacy skills when examining the impact of touchscreen device use. While prior studies highlight the role of content quality and parental involvement in touchscreen impacts on literacy, our study focused on the device type used. This strategic choice aimed to decipher the foundational link between device and literacy. Yet, the richness of content and parental engagement can significantly influence outcomes. While not the focus of this study, they are crucial for future research. We advocate for future studies to combine device type with content and parental engagement for a fuller understanding of children’s digital literacy.

Our study, forming part of the larger longitudinal research by Thongseiratch et al., 2021, shares similar limitations which can be outlined more succinctly. First, the study was constrained by the literacy tests available in Thailand, limiting our assessment to word reading and spelling from dictation, thereby possibly not capturing the full spectrum of literacy skills. Second, the evaluation of phonological awareness was conducted solely through initial sound matching, which may not fully represent all phonological awareness skills. Third, our study’s approach to RAN focused only on letter naming, potentially not the most appropriate for kindergarten-level children who may not clearly identify or articulate letters. Furthermore, a significant limitation was the lack of comprehensive measurement of screen exposure, particularly television watching in the non-use group, which could have differentially impacted language skills. Additionally, the study did not ascertain the specific types of applications used on touchscreen devices, a factor crucial in understanding the impact on emergent literacy. Finally, the disparity in the characteristics of the main caregivers between the use and non-use groups, especially in terms of education level, suggests socioeconomic factors may have influenced our findings, a crucial consideration in the context of a developing country like Thailand. These outlined limitations emphasize the need for future research to adopt a more holistic approach in evaluating the effects of touchscreen device usage on early literacy [34].

## Conclusion

Our study found that emergent literacy skills, including phonological awareness, rapid automatized naming, and letter naming scores, were highest among children who exclusively used tablets. However, after adjusting for primary caregiver characteristics, the relationship between touchscreen device use and emergent literacy did not reach statistical significance. Future research should explore the relationship between primary caregiver characteristics and a child’s emergent literacy skills, as well as the indirect and direct effects of primary caregiver education level on a child’s language and literacy development. It is also essential to examine the potential impact of touchscreen device use on preschool children’s cognitive and developmental outcomes.

## Data Availability

The datasets used and/or analysed during the current study are available from the corresponding author on reasonable request.
